# Prevalence of submicroscopic malaria infection in immigrants living in Spain

**DOI:** 10.1186/s12936-019-2870-3

**Published:** 2019-07-17

**Authors:** Isabel Fradejas, José Miguel Rubio, Ariadna Martín-Díaz, Juan María Herrero-Martínez, José Manuel Ruiz-Giardin, Gerardo Rojo-Marcos, María Velasco, María Calderón-Moreno, José Manuel Azcona-Gutierrez, Francisco Jesús Merino, Belén Andrés Olmo, María Espinosa, María Cuadrado, Esther González-Monte, Jerónimo Jaqueti, Juan Cuadros, Carolina Campelo, Alberto Delgado-Iribarren, Pablo Martín-Rabadán, Concepción García-García, María Ángeles Martín-Laso, Beatriz Valle-Borrego, María Coral García, Manuel Lizasoaín, Ana Pérez-Ayala

**Affiliations:** 1 Department of Clinical Microbiology, Hospital Universitario 12 de Octubre, Madrid, Spain; 2 National Microbiology Centre, Instituto de Salud Carlos III, Majadahonda, Madrid, Spain; 3 Internal Medicine Service, Hospital Universitario 12 de Octubre, Madrid, Spain; 4 Hospital Universitario de Fuenlabrada, Madrid, Spain; 5 Hospital Príncipe de Asturias, Alcalá de Henares, Madrid, Spain; 6 Hospital Universitario Fundación de Alcorcón, Madrid, Spain; 7 Hospital General Universitario Gregorio Marañón, Madrid, Spain; 8 Hospital de San Pedro, Logroño, La Rioja Spain; 9 Hospital Severo Ochoa, Leganés, Madrid, Spain; 10 Hospital Universitario de Getafe, Getafe, Madrid, Spain; 11 Emergency Service, Hospital Universitario 12 de Octubre, Madrid, Spain; 12 Nephrology and Renal Transplant Service, Hospital Universitario 12 de Octubre, Madrid, Spain; 13 Infectious Diseases Service Hospital Universitario 12 de Octubre, Madrid, Spain

**Keywords:** *Plasmodium*, Afebrile malaria, Submicroscopic parasitaemia, Immigrant, Tropical medicine

## Abstract

**Background:**

The importance of submicroscopic malaria infections in high-transmission areas could contribute to maintain the parasite cycle. Regarding non-endemic areas, its importance remains barely understood because parasitaemia in these afebrile patients is usually below the detection limits for microscopy, hence molecular techniques are often needed for its diagnosis. In addition to this, the lack of standardized protocols for the screening of submicroscopic malaria in immigrants from endemic areas may underestimate the infection with *Plasmodium* spp. The aim of this study was to assess the prevalence of submicroscopic malaria in afebrile immigrants living in a non-endemic area.

**Methods:**

A prospective, observational, multicentre study was conducted. Afebrile immigrants were included, microscopic observation of Giemsa-stained thin and thick blood smears, and two different molecular techniques detecting *Plasmodium* spp. were performed. Patients with submicroscopic malaria were defined as patients with negative blood smears and detection of DNA of *Plasmodium* spp. with one or both molecular techniques. Demographic, clinical, analytical and microbiological features were recorded and univariate analysis by subgroups was carried out with STATA v15.

**Results:**

A total of 244 afebrile immigrants were included in the study. Of them, 14 had a submicroscopic malaria infection, yielding a prevalence of 5.7% (95% confidence interval 3.45–9.40). In 71.4% of the positive PCR/negative microscopy cases, *Plasmodium falciparum* alone was the main detected species (10 out of the 14 patients) and in 4 cases (28.6%) *Plasmodium vivax* or *Plasmodium ovale* were detected. One patient had a mixed infection including three different species.

**Conclusions:**

The prevalence of submicroscopic malaria in afebrile immigrants was similar to that previously described in Spain. *Plasmodium vivax* and *P*. *ovale* were detected in almost a third of the submicroscopic infections. Screening protocols for afebrile immigrants with molecular techniques could be useful for a proper management of these patients.

**Electronic supplementary material:**

The online version of this article (10.1186/s12936-019-2870-3) contains supplementary material, which is available to authorized users.

## Background

Malaria remains the most important parasitic infection for humans, causing about 2000 deaths per day, especially in African children younger than 5 years old [1]. An estimated 216 million cases of malaria were reported worldwide in 2017, being the African Region the most affected area, with 90% of the documented *Plasmodium* infections [2]. Although the number of deaths had decreased until 2015, over the last 3 years, this number has remained stable [2], hence *Plasmodium* infections, and especially those caused by *Plasmodium falciparum* are still one of the most important tropical health problems.

Outside endemic areas, imported clinical malaria is a well-known disease; in 2017 more than 8000 imported malaria cases were reported in immigrants and travellers in European Union countries [3], of which 748 occurred in Spain [4]. In this regard, immigrants, including newly arrived immigrants and immigrants visiting friends and relatives (VFRs), account for most of the imported malaria cases in developed countries [5]. They often have milder symptoms and lower parasite loads than recently arrived travellers due to their semi-immunity. This immune response is a result of continuous exposure to malaria in the past and plays a key role in controlling the infection and protecting them from clinical disease [6, 7]. While management of clinical malaria is well-established in protocols in several developed countries [8, 9], submicroscopic infections are not usually discussed.

In endemic countries, including high-level and low-level transmission areas, low-density infections seems to be important for the maintenance of malaria transmission [10–12], as they can act as silent reservoirs of *Plasmodium* spp. [13–15]. Using mathematical models, the importance of treating asymptomatic population in the global control of the infection rather than only focusing on symptomatic patients has been postulated [16]. Some authors raise the issue of potential reintroduction of malaria in non-endemic areas, such as Spain where the vector is present [17], but whether submicroscopic infections have a role to play in the epidemiology of malaria in non-endemic countries remains unclear. Also, the prevalence of submicroscopic malaria among immigrants is unknown, and it is not clear if systematic screening or systematic treatment of immigrants from endemic areas would be necessary [18].

Furthermore, in addition to the importance of diagnosing mixed infections with potentially latent species like *Plasmodium vivax* and *Plasmodium ovale,* some studies suggest that submicroscopic cases are more likely to have lower levels of platelets and haemoglobin than non-infected patients, as well as higher rates of malnutrition, anaemia and thrombocytopaenia [19–21].

As for laboratory diagnostic methods, while microscopic observation of thick and thin blood smears and rapid diagnostic tests (RDTs) remain the most extended diagnostic techniques, they are insufficiently sensitive in patients with a low level parasitaemia. Hence, polymerase chain reaction (PCR) has proved to be an important tool not only to differentiate *Plasmodium* species observed in microscopy smears but also to diagnose the above-mentioned patients in recent and long-term immigrants [22].

The aim of this study was to estimate the prevalence of submicroscopic malaria infection among afebrile immigrants in Spain as well as to describe the demographical, clinical and analytical features of these patients.

## Methods

### Study design

A prospective, observational, multicentre study was conducted. Patients were enrolled at different settings in Madrid (Hospital Universitario 12 de Octubre, Hospital Universitario de Fuenlabrada, Hospital Universitario Príncipe de Asturias, Hospital Universitario Gregorio Marañón, Hospital Universitario Severo Ochoa, Hospital Universitario de Getafe and Hospital Universitario Fundación Alcorcón) and La Rioja, Spain (Hospital de San Pedro) between January 2015 and February 2018.

### Participants

Patients from endemic malaria countries (according to CDC Yellow Book criteria [23] attending Internal Medicine, Infectious/Tropical Diseases and Emergency Services of the above-mentioned hospitals, with and without clinical suspicion of malaria were initially evaluated for inclusion in the study. They were all routinely tested for malaria (diagnostic routine testing is explained in “Diagnostic methods” section). Patients without parasites in the blood smears were eligible for the study and those who agreed to be included were provided with a written informed consent.

### Inclusion criteria

All following criteria had to be fulfilled:

1. Age over 18 years-old.
2. No travels to malaria endemic areas in the month before inclusion.
3. No malaria prophylaxis intake in the month before inclusion.
4. Absence of reported fever or measured axillary temperature above 37.0 °C in the previous 24 h.

### Diagnostic methods

A total volume of 5 ml venipuncture blood was collected from all included patients and routinely processed at each setting. Microscopic observation of Giemsa-stained thin and thick blood smears were routinely performed by well-trained staff at each centre. All samples were also sent to the Microbiology Service of Hospital Universitario 12 de Octubre where thick and thin smears were observed by the same microscopist for all samples and a commercial real-time PCR (FTD-Malaria, Fast-Track Diagnostics) detecting *Plasmodium* spp. was performed. When this PCR was positive, a differential real-time PCR detecting *Plasmodium malariae, P*. *falciparum, P*. *vivax* and *P*. *ovale* was performed (FTD Malaria Differentiation, Fast-Track Diagnostics). In addition to this, blood samples were sent to the National Centre of Microbiology (Insitituto de Salud Carlos III, Majadahonda, Spain) where a multiplex nested PCR detecting the four *Plasmodium* species was performed to validate the commercial PCR (results already published) [24].

### Patient classification

Patients were classified according to a composite gold-standard reference method [25]. Patients with submicroscopic malaria were defined as patients with negative blood smears and detection of deoxyribonucleic acid (DNA) of *Plasmodium* spp. with one or both molecular techniques. When discordant results were obtained with both PCRs, patients with a positive result in at least one PCR were considered cases of submicroscopic malaria. Anti-malarial treatment was offered to all submicroscopic malaria cases. Malaria was ruled out when both molecular techniques were negative.

### Statistical analysis

Socio-demographical, epidemiological, analytical and clinical features were recorded using Redcap (Research Electronic Data Capture) software. One clinician from each hospital had access to the Redcap and was in charge of the database.

Leukopaenia was defined as < 3 × 10^3^ leukocytes/mm^3^, thrombocytopaenia was defined as < 150 × 10^3^ platelets/mm^3^ and anaemia was defined as haemoglobin level below 13 mg/dl for men and below 12 mg/dl for women. Submicroscopic malaria prevalence was calculated as number of patients with submicroscopic malaria/total number of patients included in the study. 95% confidence interval for uncertainty was calculated.

Analysis by subgroups was carried out following a univariate analysis to evaluate association of socio-demographical, clinical and analytical variables. Kolmogorov–Smirnov and Shapiro–Wilk tests were performed to assess the normal distribution of the analysed variables. Student’s t-test and Chi-square test were performed for continuous and categorical variables with normal distribution. Mann Whitney U-test and Fisher’s exact tests were performed when variables did not fit normal distribution.

Data analysis was performed with STATA v15 for macOS (StataCorp, Texas, USA).

## Results

**Fig. 1:**
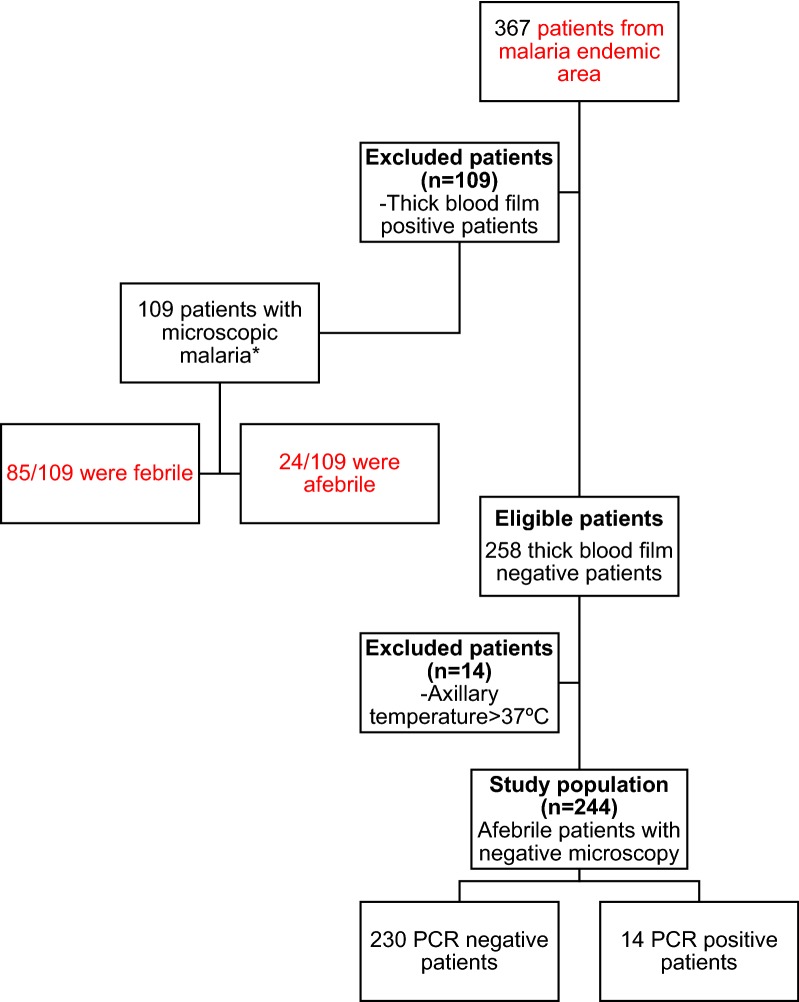
Flow-chart diagram of the total included patients. *Sociodemographic, analytical and clinical features of these patients are described in additional files

Of the 367 initially evaluated patients, 109 (29.7%) had a positive blood smear and 24/109 (22.0%) were afebrile. These 109 patients were excluded from the present study, but their socio-demographic features are detailed in Additional file 1: Table S1, Additional file 2: Table S2, Additional file 3: Table S3 and Additional file 4: Table S4. A total of 244 patients were included in the study (Fig. 1), 132 (54.1%) were male and median age at the time of consultation was 41 years (interquartile range [IQR]: 33–48). The majority (224: 91.8%) was from sub-Saharan Africa, being Equatorial Guinea and Mali the main countries of origin (33.3 and 29.3%, respectively); median time of residence in Spain until consultation was 11.1 years (IQR: 5.7–16.2). Eighty-six (35.3%) had returned at least once to an endemic area since first arrival in Spain and 60 of these 86 patients had traveled to endemic area in the year before consultation. Only 14 of these 86 (16.3%) patients that had returned to endemic area had taken anti-malarial chemoprophylaxis. Median time since last travel to an endemic area, in those who had not taken anti-malarial chemoprophylaxis was 6.2 months (IQR: 3.5–9.4). Of the 244 patients, 104 (57.1%) referred a previous episode of malaria. Socio-demographic, epidemiological, clinical and analytical features of the total population and univariate analysis by subgroups are shown in Tables 1, 2, 3 and 4.

**Table 1:** Socio-demographic features of the total 244 patients and univariate analysis by subgroups

Frequency, n (%)	Total N = 244	Patients with submicroscopic malaria N = 14	Negative patients N = 230	*P* value*
Age [years], median (IQR^a^)	41 (33–48)	41 (32–45)	41 (23–48)	0.742
Gender, male, n (%)	132 (54.1%)	8 (57.1%)	124 (53.9%)	1.000
Sub-Saharan origin, n (%)	224 (91.8%)	14 (100%)	210 (91.3%)	1.000
Time of residence in Spain [years], median (IQR)	11.1 (5.7–16.2)	8.9 (4.3–12.9)	11.3 (6.0–16.3)	0.163
Travels to endemic area since first arrival in Spain (yes/no), n (%)	86 (35.3)	7 (50.0)	79 (34.4)	0.257
Anti-malarial chemoprophylaxis^a^, n (%)	14/86 (16.3)	1/7 (14.3)	13/79 (16.5)	1.000
Time from last travel to endemic area^b^ [months], median (IQR)	6.2 (3.5–9.4)	4.0 (3.4–6.0)	6.6 (3.5–9.5)	0.170
Previous malaria, n (%)	104 (42.6)	6 (42.9)	98 (42.6)	0.160

*P values < 0.05 were considered statistically significant. Mann Whitney U-test and Fischer’s exact tests were performed for quantitative and categorical variables respectively

^a^Patients who had returned to endemic area after first arrival in Spain were defined as the total population (denominator)

^b^Evaluated in patients without chemoprophylaxis intake before last time to endemic area

**Table 2:** Laboratory test results of the total 244 patients and univariate analysis by subgroups

	Total N = 244	Patients with submicroscopic malaria N = 14	Negative patients N = 230	*P* value*
Leukocyte count (× 10^3^/mm^3^), median (IQR)	5.3 (4.0–6.9)	5.2 (3.9–7.4)	5.3 (4.0–6.9)	0.898
Leukopaenia^a^, n (%)	55 (22.5)	4 (28.6)	51 (22.2)	0.525
Platelet count (× 10^3^ /mm^3^), median (IQR)	219 (172–264)	204 (177–243)	219 (172–266)	0.459
Thrombocytopaenia^b^, n (%)	33 (13.52)	3 (21.4)	30 (13.0)	0.413
Haemoglobin level (mg/dl), median (IQR)	13.4 (12.1–14.6)	14.0 (12.2–15.0)	13.4 (12.1–14.5)	0.361
Anaemia^c^, n (%)	65 (26.6)	3 (21.4)	62 (26.9)	0.765

*P values < 0.05 were considered statistically significant. Mann Whitney U-test and Fisher’s exact tests were performed for quantitative and categorical variables, respectively

^a^Defined as < 3 × 10^3^ leukocytes/mm^3^

^b^Defined as < 150 × 10^3^ platelets/mm^3^

^c^Defined as < 13 mg haemoglobin/dl for men and < 12 mg haemoglobin/dl for women

**Table 3:** Reason for consultation of the total 244 patients and univariate analysis by subgroups

Frequency, n (%)	Total N = 244	Patients with submicroscopic malaria N = 14	Negative patients N = 230	*P* value*
Immigrant screening	39 (16.0)	6 (42.9)	33 (14.4)	*0*.*013*
HIV follow-up	34 (13.9)	3 (21.4)	31 (13.5)	0.422
Abdominal pain	27 (11.7)	0	27 (11.7)	0.376
Asthenia	18 (7.4)	3 (21.4)	15 (6.5)	0.073
Headache	18 (7.4)	2 (14.3)	16 (7.0)	0.276
General discomfort	11 (4.5)	1 (7.1)	10 (4.4)	0.485
Eosinophilia	7 (2.9)	1 (7.1)	6 (2.6)	0.342
Myalgia	6 (2.5)	2 (14.3)	4 (1.7)	*0*.*040*
Others	92 (37.7)	2 (14.3)	90 (39.1)	0.087

*P values < 0.05 were considered statistically significant (in italics). Mann Whitney U-test and Fisher’s exact tests were performed for quantitative and categorical variables respectively

**Table 4:** Co-morbidities and other infectious diseases in the 244 afebrile patients and univariate analysis of subgroups

Frequency, n (%)	Total N = 244	Patients with submicroscopic malaria N = 14	Negative patients N = 230	*P* value*
Co-morbidities	102 (41.8)	4 (28.6)	98 (42.6)	0.406
Diabetes Mellitus	16 (6.6)	1 (7.1)	15 (6.5)	1.000
Arterial hypertension	24 (9.8)	2 (14.3)	22 (9.6)	0.635
Dyslipidaemia	14 (5.7)	0	14 (6.1)	1.000
Neoplasia	8 (3.3)	0	8 (3.5)	1.000
Transplantation	3 (1.2)	0	3 (1.3)	1.000
Other comorbidities	72 (30.8)	2 (15.4)	70 (31.7)	0.354
Infectious diseases				
HIV	61 (25.0)	4 (28.6)	57 (24.8)	0.754
HBV	34 (13.9)	2 (14.3)	32 (13.9)	1.000
HCV	7 (2.9)	1 (7.1)	6 (2.6)	0.342
Tuberculosis	20 (8.2)	1 (7.1)	19 (8.3)	1.000
*Strongyloides stercoralis*	14 (5.7)	2 (14.3)	12 (5.2)	0.187
Filariasis	6 (2.5)	2^a^ (14.3)	4^b^ (1.7)	*0.040*
Schistosomiasis	9 (3.7)	2 (14.3)	7 (3.0)	0.087
Intestinal parasites	8 (3.3)	1 (7.1)	7 (3.0)	0.381

*P values < 0.05 were considered statistically significant (in italics). Mann Whitney U-test and Fisher’s exact tests were performed for quantitative and categorical variables, respectively

^a^One *Mansonella perstans* and one lymphatic filariasis (Only serological diagnosis)

^b^*Onchocerca* spp.

Fourteen patients were defined as submicroscopic malaria cases by PCR, yielding a prevalence of 5.7% (95% confidence interval 3.45–9.40). In 230 patients (94.3%) all microbiological tests for malaria were negative. Although the associations were not statistically significant, submicroscopic malaria cases had shorter time of residence in Spain than negative patients (8.9 years in submicroscopic malaria cases vs 11.3 years in negative patients, *P* = 0.163) as well as shorter time since last travel to endemic area (4.0 months in submicroscopic malaria cases vs 6.6 months in negative patients, *P* = 0.170). Immigrant screening (42.9% in submicroscopic malaria cases vs 14.4% in negative patients) and myalgia (14.3% in submicroscopic malaria cases vs 1.7% in negative patients) were the reasons for consultation associated to submicroscopic malaria cases (*P* = 0.013 and *P* = 0.040). Filariasis was also associated with submicroscopic malaria patients (14.3% in submicroscopic malaria vs 1.7% in negative patients *P* = 0.040). Higher percentages of strongyloidiasis and schistosomiasis were found in submicroscopic malaria patients although these associations were not significant (14.3% vs 5.2%, *P* = 0.187 and 14.3% vs 3.0%, *P* = 0.087, respectively).

The main reason for consultation of the 14 submicroscopic patients was immigrant screening, representing the 15% (6/39) of all patients attending the immigrant screening consultation. Among women, there was one who was pregnant. Socio-demographic, epidemiological, analytical and microbiological features of the 14 patients with submicroscopic malaria are shown in Table 5.

**Table 5:** Descriptive features of the 14 submicroscopic malaria cases

Gender	Age (years)	Area of origin	Time of residence in Spain (years)	Travels to endemic area in the year before inclusion	Time from the last travel to endemic area (months)	Reason for consultation	Antimalarial chemoprophylaxis prior last travel to endemic area	Previous malaria	Detected species
Male	31	Africa (Mali)	9.5	Yes	7.4	Immigrant screening	No	No	*Plasmodium falciparum*
Male	42	Africa (Nigeria)	16.5	Yes	4.5	Headache, myalgia, others (cough and dyspnea)	Unknown	Yes	*Plasmodium falciparum*
Male	46	Africa (Equatorial Guinea)	4.3	Yes	5.1	Asthenia	Unknown	Unknown	*Plasmodium falciparum*
Female	44	Africa (Equatorial Guinea)	12.9	No	Unknown	Immigrant screening	–	Yes	*Plasmodium falciparum*
Female	32	Africa (Equatorial Guinea)	12.8	Yes	3.7	Asthenia	No	Yes	*Plasmodium falciparum*
Female	50	Africa (Equatorial Guinea)	8.9	Yes	2	Asthenia, headache, general discomfort, myalgia	No	Unknown	*Plasmodium falciparum*
Male	31	Africa (Eritrea)	0.3	No	Unknown	Immigrant screening	–	Unknown	*Plasmodium vivax*
Male	18	Africa (Equatorial Guinea)	0.4	No	Unknown	Immigrant screening	–	No	*Plasmodium falciparum* + *Plasmodium malariae* + *Plasmodium ovale*
Female	45	Africa (Equatorial Guinea)	7.6	Yes	6.0	Immigrant screening and eosinophilia	No	Unknown	*Plasmodium falciparum*
Male	40	Africa (Nigeria)	16.4	Yes	8.0	HIV follow-up	Yes	Yes	*Plasmodium falciparum*
Male	42	Africa (Nigeria)	14.2	No	–	Immigrant screening	–	Unknown	*Plasmodium falciparum*
Male	40	Africa (Guinea Bissau)	–	Unknown	–	HIV follow-up	–	Yes	*Plasmodium vivax*
Female	62	Africa (Equatorial Guinea)	–	No	–	Others (low back pain)	–	Unknown	*Plasmodium falciparum* + *Plasmodium ovale*
Female	39	Africa (Senegal)	8.7	No	–	HIV follow-up	–	Yes	*Plasmodium falciparum*

Ten patients (71.4%) had *P*. *falciparum* submicroscopic malaria, 2 (14.3%) patients had mixed infections (1 *P*. *falciparum *+ *P*. *ovale* and 1 *P*. *falciparum*, *P*. *malariae* + *P*. *ovale*) and 2 (14.3%) patients had *Plasmodium vivax* infection.

## Discussion

In Spain, scarce studies have evaluated the prevalence of submicroscopic malaria in afebrile immigrants [26, 27]. These authors describe slightly different levels of prevalence than the present study, (4.6% and 8.9% vs 5.7%). The different performances of the molecular techniques, the chosen reference methods (simple gold-standard vs composite gold-standard), the design of the study (single-centre vs multicentre) and the inclusion criteria (the definition of “asymptomatic” and the minimal time of residence in Spain vary among these studies) are important features that could explain the differences in these prevalence levels.

According to some studies from endemic areas up to 50% of sub-clinical children carrying *P*. *falciparum*, could develop clinical malaria [28]. Currently there are no studies that assess the risk of developing clinical malaria in adult immigrants arriving to non-endemic areas, although some authors suggest that few afebrile adults develop symptoms after a relatively short time period [29]. In this study, anti-malarial treatment was offered to all diagnosed patients and follow-up visits were not registered in the database, so the risk of clinical relapse was not evaluated. Submicroscopic malaria caused by *P*. *falciparum* was detected in three patients who had not returned to endemic area in the year before consultation, noting the possibility of being pauci or asymptomatic for a long period, as described by other authors [30]. Unfortunately, due to the lack of epidemiological data, differences among the period of incubation of submicroscopic *P*. *falciparum* infection and mixed or non-falciparum infections were not evaluated.

Regarding analytical features, evidence from studies developed in sub-Saharan countries associates anaemia and thrombocytopaenia not only with symptomatic but also with afebrile patients with malaria [19]. In the study cohort, 3 of the 14 afebrile patients had anaemia and thrombocytopaenia but there was not statistically significant association, probably because of the low number of submicroscopic malaria cases, there is nothing to conclude about this. Further studies in non-endemic countries would be interesting on this matter. Nevertheless, statistically significant association of submicroscopic malaria with myalgia was described, which to date, has been described with febrile malaria but not with afebrile patients. The association with filarial infection has also been observed in another study, suggesting that the modulation in the immune response produced by filariasis may protect against clinical malaria [27]. More studies are needed to better understand the interactions among these parasites and the immune system.

In non-endemic areas, where public health programmes have eradicated the infection, the importance of afebrile carriers also relies on the possibility of reintroduction of the infection. Although to date, the reintroduction of malaria in Europe is unlikely [31], several cases and outbreaks of autochthonous malaria have been described in the last decade [32–34]. *Anopheles maculipennis* complex (*Anopheles atroparvus* being the most frequent species) is widely distributed throughout Europe and especially Spain [35, 36], hence the potential reintroduction of *P*. *vivax* (which is the only malaria species that have proved to be transmitted by *An*. *atroparvus* [37]), cannot be completely ruled out. Two of the submicroscopic cases detected in the present study were *P*. *vivax* infections, in contrast with other Spanish studies describing *P*. *falciparum* infections or mixed infections only with *P*. *malariae* and *P*. *ovale* [26, 27]. Although it is a small number, they only represent a small percentage of all *P*. *vivax* infections since not all febrile immigrants were included, so the possibility of autochthonous transmission of malaria should be kept in mind and epidemiological and entomological vigilance should not be overlooked.

Treatment protocols for asymptomatic patients before arriving to non-endemic countries have been developed in some countries, including the USA [38]. The US Refugee Health Guidelines from 2012 [39] recommended preemptive treatment for malaria in refugees using artemether/lumefantrine, which only is effective against blood-stage parasites; hence the risk of relapses caused by the dormant liver stages of *P*. *ovale* and *P*. *vivax* remains a potential problem, and noting that, as in this cohort, an important percentage of afebrile infections can be caused by these species. In this study, 15% of asymptomatic immigrants attending an immigrant screening consultation had submicroscopic malaria, the revision and actualization of these protocols should be a priority, mainly for those with anaemia and thrombocytopaenia and for those asymptomatic or with atypical symptoms like myalgias.

The main limitation of this study was the low number of submicroscopic cases detected, limiting the possibility of statistical analysis, and the lack of epidemiological information in some of the cases because of the existing sociocultural and language barrier. It is surprising that only 43% of submicroscopic cases reported having previous malaria infections, suggesting that it could probably be due to the difficulty in communication or due to a lack of awareness of the disease. The population included in the study represents a wide range of the immigrant population living in Madrid, as it is a multicentre study with a large number of hospitals participating in it. Although, only one hospital outside Madrid is included, so maybe a broader study could recall more reliable data.

## Conclusion

The prevalence of submicroscopic imported malaria found in this study was similar to that previously described by other authors in non-endemic areas in afebrile immigrants, namely *P*. *vivax* and *P*. *ovale* were involved in an important percentage of these infections. Screening protocols for afebrile immigrants with molecular techniques could be useful not only for a proper management and treatment of submicroscopic infections but also to prevent the potential reintroduction of malaria in areas where the vector is present.

## Additional files

**Additional file 1: Table S1.** Socio-demographic features of the total 109 microscopy positive patients.

**Additional file 2: Table S2.** Laboratory test results of the 109 patients with microscopic malaria.

**Additional file 3: Table S3.** Reason for consultation of the total 109 patients with microscopic malaria.

**Additional file 4: Table S4.** Co-morbidities and other infectious diseases of the 109 patients with microscopic malaria.

## Data Availability

All data generated and analysed during the study are available from the corresponding author on a reasonable request.

## Abbreviations

DNA: deoxyribonucleic acid
RDTs: rapid diagnostic tests
PCR: polymerase chain reaction
VFRs: visiting friends and relatives
