# Supplementary material for: Prevalence of submicroscopic malaria infection in immigrants living in Spain
Source: Malar J. 2019 Jul 17;18:242. doi: 10.1186/s12936-019-2870-3 (PMC6637620; doi:10.1186/s12936-019-2870-3)
Supplement: Supplementary file 1 — Additional file 1: Table S1. Socio-demographic features of the total 109 microscopy positive patients. [file 12936_2019_2870_MOESM1_ESM.docx]

Additional files

**Table S1: Socio-demographic features of the total 109 microscopy positive patients.**

| **Frequency, n (%)** | **Total**  **N=109** |
| --- | --- |
| **Age [years], median (IQR*)** | 39 (31-46) |
| **Gender, male, n (%)** | 62 (56.9) |
| **African origin, n (%)** | 104 (96.3) |
| **Time of residence in Spain [years], median (IQR)** | 9.9 (0.1-15.3) |
| **Travels to endemic area since first arrival in Spain (yes/no),**  **n (%)** | 70 (64.2) |
| **Antimalarial chemoprophylaxis**, n (%)** | 4/70 (5.7) |
| **Time from last travel to endemic area [months], median (IQR)** | 0.36 (0.19-1.02) |
| **Previous malaria, n (%)** | 51 (46.8) |
| **Detected species in the present admission** |  |
| ***Plasmodium falciparum*, n (%)** | 91 (83.9) |
| **Mixed infections, n (%)** | 12 (11.0)*** |
| ***Plasmodium ovale,* n (%)** | 3 (2.8) |
| ***Plasmodium malariae,* n (%)** | 2 (1.8) |
| ***Plasmodium vivax,* n (%)** | 1 (0.6) |

* IQR: Interquartile range

**Patients who had returned to endemic area after first arrival in Spain were defined as the total population (denominator)

***8 were infections by *Plasmodium falciparum* and *Plasmodium ovale* and 4 were mixed infections by *Plasmodium falciparum* and *Plasmodium malariae.*
