# Supplementary material for: Prevalence of submicroscopic malaria infection in immigrants living in Spain
Source: Malar J. 2019 Jul 17;18:242. doi: 10.1186/s12936-019-2870-3 (PMC6637620; doi:10.1186/s12936-019-2870-3)
Supplement: Supplementary file 2 — Additional file 2: Table S2. Laboratory test results of the 109 patients with microscopic malaria. [file 12936_2019_2870_MOESM2_ESM.docx]

**Table S2: Laboratory test results** **of the 109 patients with microscopic malaria.**

|  | **Total**  **N=109** |
| --- | --- |
| **Leukocyte count (x10^3^ /mm^3^), median (IQR)** | 5.0 (4.1-6.5) |
| **Leukopaenia*, n (%)** | 27 (24.8) |
| **Platelet count (x10^3^ /mm^3^), median (IQR)** | 107 (79-143) |
| **Thrombocytopaenia**, n (%)** | 85 (78.0) |
| **Haemoglobin level (mg/dl), median (IQR)** | 13.0 (11.6-14.3) |
| **Anaemia***, n (%)** | 43 (39.5) |

*Defined as <3x10^3^ leukocytes/mm^3^

**Defined as <150x10^3^ platelets/mm^3^

***Defined as <13 mg hemoglobin/dl for men and < 12 mg hemoglobin/dl for women
