# Supplementary material for: Prevalence of submicroscopic malaria infection in immigrants living in Spain
Source: Malar J. 2019 Jul 17;18:242. doi: 10.1186/s12936-019-2870-3 (PMC6637620; doi:10.1186/s12936-019-2870-3)
Supplement: Supplementary file 3 — Additional file 3: Table S3. Reason for consultation of the total 109 patients with microscopic malaria. [file 12936_2019_2870_MOESM3_ESM.docx]

**Table S3: Reason for consultation of the total 109 patients with microscopic malaria.**

| **Reason for consultation** | **Frequency, n (%)**  **N=109** |
| --- | --- |
| **Fever** | 85 (78.0) |
| **Headache** | 44 (40.4) |
| **General discomfort** | 32 (29.4) |
| **Myalgia** | 24 (22.0) |
| **Shivers** | 22 (20.2) |
| **Nausea** | 20 (18.4) |
| **Arthralgia** | 21 (19.3) |
| **Asthenia** | 20 (18.4) |
| **Diarrhea** | 16 (14.7) |
| **Abdominal pain** | 15 (13.8) |
| **Vomiting** | 15 (13.8) |
| **Cough** | 11 (10.9) |
| **Sweats** | 6 (5.5) |
| **Dizziness** | 6 (5.5) |
| **Weakness** | 5 (4.6) |
| **Dysuria** | 5 (4.6) |
| **Thrombocytopaenia** | 4 (3.7) |
| **Dyspnea** | 3 (2.8) |
| **Thoracic pain** | 3 (2.8) |
| **Anaemia** | 1 (0.9) |
| **Jaundice** | 1 (0.9) |
| **Leukopaenia** | 1 (0.9) |
| **Others** | 17 (15.6) |
