# Supplementary material for: Prevalence of submicroscopic malaria infection in immigrants living in Spain
Source: Malar J. 2019 Jul 17;18:242. doi: 10.1186/s12936-019-2870-3 (PMC6637620; doi:10.1186/s12936-019-2870-3)
Supplement: Supplementary file 4 — Additional file 4: Table S4. Co-morbidities and other infectious diseases of the 109 patients with microscopic malaria. [file 12936_2019_2870_MOESM4_ESM.docx]

**Table S4: Co-morbidities and other infectious diseases of the 109 patients with microscopic malaria.**

| **Variable** | **Frequency, n (%), N=109** |
| --- | --- |
| **Co-morbidities** |  |
| Arterial hypertension | 4 (3.7) |
| Neoplasia | 3 (2.8) |
| Diabetes Mellitus | 2 (1.8) |
| Dyslipidaemia | 2 (1.8) |
| Transplantation | 1 (0.9) |
| Other comorbidities | 9 (9.3) |
| **Other Infectious diseases** |  |
| HIV | 11 (10.1) |
| Filariasis | 4 (3.7) |
| HBV | 3 (2.8) |
| Tuberculosis | 3 (2.8) |
| HCV | 2 (1.8) |
| *Strongyloides stercoralis* | 2 (1.8) |
| Schistosomiasis | 1 (0.9) |
| Intestinal parasites | 1 (0.9) |
